# Decomposition of Composite Electric Field in a Three-Phase D-Dot Voltage Transducer Measuring System

**DOI:** 10.3390/s16101683

**Published:** 2016-10-12

**Authors:** Xueqi Hu, Jingang Wang, Gang Wei, Xudong Deng

**Affiliations:** 1 State Key Laboratory of Power Transmission Equipment & System Security and New Technology, Chongqing University, Chongqing 400044, China; 20141102019@cqu.edu.cn; 2 Maintenance Branch of State Grid Chongqing Electric Power Company, Chongqing 400039, China; cqweig@163.com (G.W.); dengxudong_023@163.com (X.D.)

**Keywords:** three-phase D-dot voltage transducer, Ansoft Maxwell, decomposition of composite field

## Abstract

In line with the wider application of non-contact voltage transducers in the engineering field, transducers are required to have better performance for different measuring environments. In the present study, the D-dot voltage transducer is further improved based on previous research in order to meet the requirements for long-distance measurement of electric transmission lines. When measuring three-phase electric transmission lines, problems such as synchronous data collection and composite electric field need to be resolved. A decomposition method is proposed with respect to the superimposed electric field generated between neighboring phases. The charge simulation method is utilized to deduce the decomposition equation of the composite electric field and the validity of the proposed method is verified by simulation calculation software. With the deduced equation as the algorithm foundation, this paper improves hardware circuits, establishes a measuring system and constructs an experimental platform for examination. Under experimental conditions, a 10 kV electric transmission line was tested for steady-state errors, and the measuring results of the transducer and the high-voltage detection head were compared. Ansoft Maxwell Stimulation Software was adopted to obtain the electric field intensity in different positions under transmission lines; its values and the measuring values of the transducer were also compared. Experimental results show that the three-phase transducer is characterized by a relatively good synchronization for data measurement, measuring results with high precision, and an error ratio within a prescribed limit. Therefore, the proposed three-phase transducer can be broadly applied and popularized in the engineering field.

## 1. Introduction

The operational status and health level of alternating-current transmission lines could be directly reflected by the amplitude and phase position of three-phase voltage, offering direct data references for the operation and maintenance of an electric system [1]. The conventional measuring method is to install a mutual voltage inductor in transformer substations of line start and end ports to detect line voltage [2]. With the continuous development of smart power grids, the need for real-time status monitoring of the line voltage along overhead transmission lines has become increasingly urgent, as has the development of intelligent early-warning and automatic control mechanism [3]. To address this problem, we designed the D-dot voltage transducer based on the electric-field coupling principle, which recognizes that non-contact measurement indeed overcomes a series of problems caused by direct contact between traditional voltage transducers and detected wires or instruments during the measuring process [4]. In addition, the non-contact measuring method decreases ferromagnetic resonance, reduces reactive power consumption, and has good dynamic response as well as small volume. This method has been proven to be a safe and convenient measurement method [1,5].

Currently, a large amount of research work has been carried out to improve the performance of field-type transducers. The authors of [6] proposed an optical voltage transducer based on the Pockels/Kerr effect to achieve the non-contact measurement for voltage. This method has high measurement accuracy, however, optical devices are expensive and have limited precision, which limits their wider, practical application. In a wider scope; authors of [7,8] proposed the non-contact voltage transducer based on the electric-field coupling principle; the transducer provides a simple structure non-contact transducer, still in prototype form. In references [9,10,11], a self-integral D-dot voltage transducer was introduced to increasingly perfect the transducer in aspects of calculation principle and structural design. These are based on the measurement of single transducer. The author of [12] considered the use of a three-phase transmission line in order to establish a D-dot voltage simulation model, and studied the distribution parameter of the three-phase voltage transducer. However, the transducer was premised on the condition that the sensor installation location would be close to the wire. In current projects, three-phase transmission lines are simultaneously measured with the condition that a safe distance is needed between the transducer and the transmission lines. With this condition, the measurement values of each transducer are the result of the simultaneous action of the three-phase transmission lines. The problem of the simultaneous synthesis of the electric field in the three-phase voltage transducer measuring system has not been studied before. Therefore, research on the three-phase D-dot voltage transducer measuring system is based on decomposition of the composite field using non-contact measurement with convenient and efficient parameters of the voltage in a transmission line.

Thus, charge simulation method is used to deduce a composite electric field equation at any point of a three-phase transmission line. This paper establishes a mathematical model for horizontally arranged lines, to decompose the composite electric field, calculate the electric field component and obtain a derived inversion equation of the electric field component. Simulation calculation software has been adopted to determine the voltage of three-phase lines by measuring the data of a three-phase transducer, and an experimental platform has been constructed to verify the performance of the studied measuring system and validate values of theoretical studies and actual systematic projects.

## 2. Detection Principle

### 2.1. Analysis of Measuring Principle for D-Dot Transducer 

The working principle of a D-dot transducer can be summarized as follows: performing non-contact indirect measurements of the voltage by measuring the rate of change for electric displacement vector [13].

Specific measurement principles can be found in references [14,15]. For the measurement of the wire, the relationship between the surrounding electric field and the conductor potential is as shown in Equation (2) and in boundary conditions as Equation (1):

(1) $$\left\{ \begin{array}{l} \varphi_{R\leq r_{0}}=\varphi_{0}(t)\\ \varphi_{R\to \infty }=0 \end{array}\right.$$

(2) $$\vec{E}(t)=\frac{\varphi_{0}(t)}{r}\ln(r_{0})\cdot {\vec{e}}_{{}_{R}}$$

where $\varphi_{0}(t)$ is represented as the measured cable potential, E(*t*) as the electric field intensity of the measured point, *r* as the distance of the measured point to the wire center, and *r*_0_ as the radius of the electric cable.

Furthermore, the resistance drop has a positive relationship with the rate of change related to the electric field in the electrode [14], which can be proven by Gauss theorem. By using Equation (2), the relationship between the detected potential and transducer output can be obtained:

(3) $$U_{0}=\frac{\varepsilon_{0}A_{eq}R}{r\ln(r_{0})}\frac{d}{dt}\varphi_{0}(t)$$

In the equation, *A_eq_* is represented as the equivalent area of the voltage transducer, whereas *R* is the load resistance value.

### 2.2. Three-Phase Electrical Field Solved by the Charge Simulation Method

Based on the uniqueness theorem of the electric field, Charge Simulation Method is to replace electric charges distributed continuously in the surface of energized conductor by a set of discrete charges in the conductor (such as set a group of point, line and ring charges), and all the charges are simulative ones [16,17]. Simulative charge values can be acquired by solving the linear algebraic group in potential boundary conditions. Thereafter, the superposition theorem can be adopted to calculate the electric potential and intensity of any position in the electric field. When the transmission conductor is parallel to the ground, if the conductor radius is much smaller than the height of the transmission line to the ground and the voltage in the conductor is three-phase symmetrical sinusoidal then the charges in the transmission conductor will be distributed evenly [18].

For the convenience of engineering calculations, the simplified treatment is conducted for the model of three-phase transmission lines: the ground is an infinitely great conductor plane with an electric potential of 0; charges have no electric field distortion. In the computational domain, the electric three-phase conductor shall only be considered and surrounding conductors can be neglected [19], such as the influence of the tower and metal fittings.

**Figure 1:**
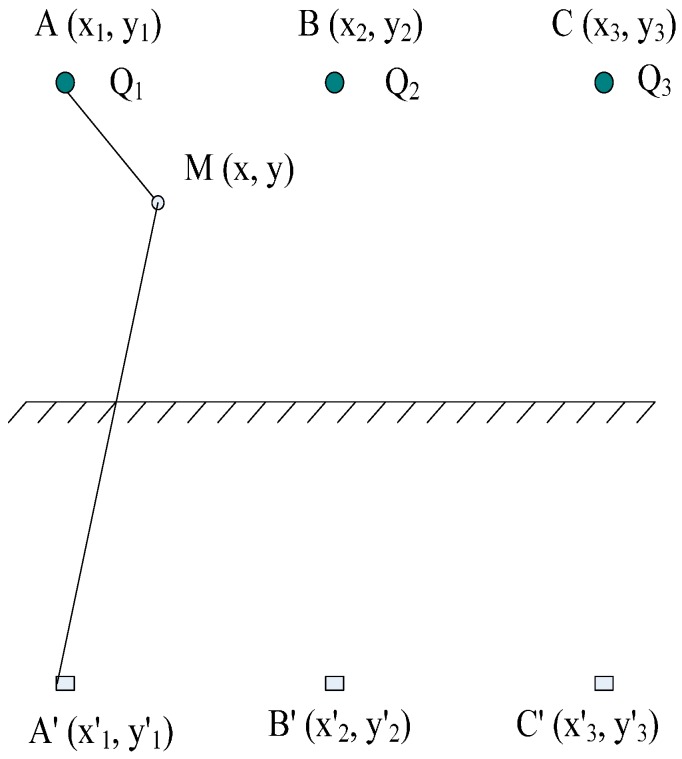
Map of the charge simulation method.

Figure 1 shows a diagram of the charge simulation method and three simulative charges Q_j_ (j = 1,2,3) that are set in the interior of the transmission conductor with three phases, namely, A, B and C. In addition, a rectangular coordinate system is established in a two-dimensional computational domain with given potential boundary conditions, and (x, y) is regarded as its coordinate. 

According to the superposition theorem, electric potential expression *Q* = *P*^−^^1^ φ can be obtained [19], which is constructed by three simulative charges set above.

(4) $$\left\{ \begin{array}{l} P_{11}Q_{1}+P_{12}Q_{2}+P_{13}Q_{3}=\varphi_{A}\\ P_{21}Q_{1}+P_{22}Q_{2}+P_{23}Q_{3}=\varphi_{B}\\ P_{31}Q_{1}+P_{32}Q_{2}+P_{33}Q_{3}=\varphi_{C} \end{array}\right.$$

In Equation (4), $P_{ii}$ is represented as the electric potential value of *j*th simulative charge generated in *i*th point; $\varphi_{A}$, $\varphi_{B}$, $\varphi_{C}$, are the voltages of the three phases in the conductor. In the *P* electric potential coefficient matrix, the *P_ij_* solution equation based on image theory is:

(5) $$\left\{ \begin{array}{l} P_{ii}=\frac{1}{2\pi \varepsilon_{0}}\ln\frac{2h_{i}}{R_{i}}\\ P_{ij}=\frac{1}{2\pi \varepsilon_{0}}\ln\frac{2{L^{'}}_{ij}}{L_{ij}},P_{ij}={P^{'}}_{ij},(i\neq j) \end{array}\right.$$

In Equation (5), $\varepsilon_{0}$ is the air dielectric coefficient; *h_i_* is the height of the conductor to the ground; R*_n_* is the radius of the conductor; ${L^{'}}_{ij}$ and $L_{ij}$ are the distance of *i* conductor and its mirror image to *j* conductor.

Based on superposition theorem, the values of the electric field intensity *E_x_* and *E_y_* respectively in the horizontal and vertical directions for any point (*x*, *y*) in the computational domain can be obtained:

(6) $$\left\{ \begin{array}{l} E_{x}=\frac{1}{2\pi \varepsilon_{0}}\sum_{i=1}^{N+n_{0}}Q_{i}(\frac{x-x_{i}}{L_{i}^{2}}-\frac{x-x_{i}}{L_{i}^{'2}})\\ E_{y}=\frac{1}{2\pi \varepsilon_{0}}\sum_{i=1}^{N+n_{0}}Q_{i}(\frac{y-y_{i}}{L_{i}^{2}}-\frac{y-y_{i}}{L_{i}^{'2}}) \end{array}\right.$$

The transducer is installed under the transmission conductor. Given that the electric field intensity in the x direction is quite small, only the y direction shall be considered, namely, the electrical field intensity in the vertical direction. According to Equation (6), the composite electric field intensity of any position near the conductor can be calculated [20]. In order to further calculate the electric field components of neighboring phases, the conductor voltage could finally be obtained by decomposing the composite electric field when the transmission conductor is arranged horizontally and backward substituting the acquired electric field components into Equation (3).

### 2.3. Calculation Example

**Figure 2:**
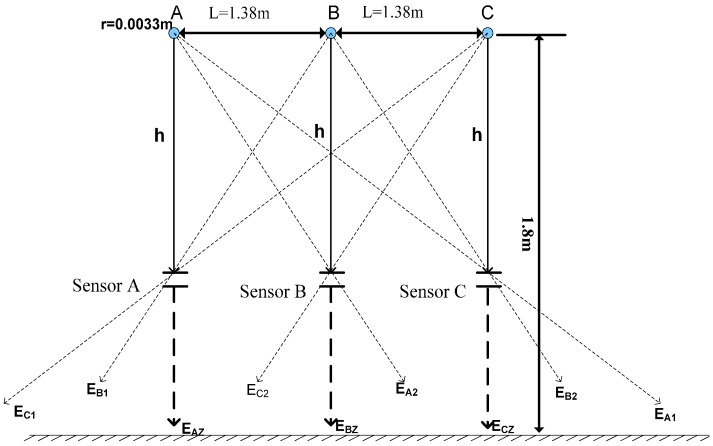
Decomposition of the electric field for the horizontally arranged line.

Taking the horizontally arranged 10 kV overhead transmission line as the case, its composite electric field detected by a three-phase transducer is decomposed for simulation calculation. Based on LGJ-500-45 regulations, the sectional area of the conductor radius is 35 mm^2^, the distance between phases is L = 1.38 m, and the vertical height from the ground is 1.8 m. Three D-dot voltage transducers are respectively installed right under the three phases of the conductor, namely, Sensor A, Sensor B and Sensor C, h = 0.15 m, as shown in Figure 2. 

In a 10 kV transmission line, a three phase transmission line is arranged horizontally, as shown in Figure 2. *E_AZ_* is the electric field component of the transducer arranged vertically under an A-phase conductor; *E_C1_* is represented as the electric field component of the C-phase conductor to Transducer A; and *E_B1_* is the electric field component of the B-phase to Transducer A. Meanwhile, the electric field components of all phases in Transducers B and C are as shown by the marks of the figure and the distances of the three transducers to the conductor are all equal to *h*. From Figure 2, the mathematical relationship between electrical field measured by the transducer and the electric field components of all phases can be obtained by taking advantage of trigonometric function, as shown below:

(7) $$\left\{ \begin{array}{l} E_{A}=E_{AZ}+\frac{E_{B1}\cdot h}{\sqrt{L^{2}+h^{2}}}+\frac{E_{C1}\cdot h}{\sqrt{4L^{2}+h^{2}}}\\ E_{B}=E_{BZ}+\frac{E_{A2}\cdot h}{\sqrt{L^{2}+h^{2}}}+\frac{E_{C2}\cdot h}{\sqrt{L^{2}+h^{2}}}\\ E_{C}=E_{CZ}+\frac{E_{B2}\cdot h}{\sqrt{L^{2}+h^{2}}}+\frac{E_{A1}\cdot h}{\sqrt{4L^{2}+h^{2}}} \end{array}\right.$$

From Equation (7), the electric field intensity of Transducer A is composed of three electric field components: A-phase conductor component and the disturbance variables of the B and C conductors. Similarly, the electric field intensity measured by Transducers B and C is respectively constituted by the electric field component of the respective conductors and disturbance variables of neighboring two-phase conductors. Equation (6) shall be substituted into Equation (7):

(8) $$\left\{ \begin{array}{l} E_{AZ}=\frac{1}{2\pi \varepsilon_{0}}\sum_{i=1}^{N+n_{0}}Q_{i}(\frac{y-y_{i}}{L_{i}^{2}}-\frac{y-y_{i}}{L_{i}^{'2}})-\frac{E_{B1}\cdot h}{\sqrt{L^{2}+h^{2}}}-\frac{E_{C1}\cdot h}{\sqrt{4L^{2}+h^{2}}}\\ E_{BZ}=\frac{1}{2\pi \varepsilon_{0}}\sum_{i=1}^{N+n_{0}}Q_{i}(\frac{y-y_{i}}{L_{i}^{2}}-\frac{y-y_{i}}{L_{i}^{'2}})-\frac{E_{A2}\cdot h}{\sqrt{L^{2}+h^{2}}}-\frac{E_{C2}\cdot h}{\sqrt{L^{2}+h^{2}}}\\ E_{CZ}=\frac{1}{2\pi \varepsilon_{0}}\sum_{i=1}^{N+n_{0}}Q_{i}(\frac{y-y_{i}}{L_{i}^{2}}-\frac{y-y_{i}}{L_{i}^{'2}})-\frac{E_{B2}\cdot h}{\sqrt{L^{2}+h^{2}}}-\frac{E_{A1}\cdot h}{\sqrt{4L^{2}+h^{2}}} \end{array}\right.$$

After the measurement, the above results in Table 1 are incorporated into Equation (8), in order to obtain the electric field strength component of the three-phase transmission line that is *E_Az_*, *E_Bz_* and *E_Cz_*. The line voltage of the three-phase transmission line can be calculated by Equations (1)–(3). When the voltage phase is 0[deg] and contrasts with the reverse generation results of the actual voltage of transmission line, the relative amplitude errors are as shown in the following table:

Based on the data from Table 2, the proposed synthesis algorithm is effective and has high precision. The algorithm for the synthesis of the field decomposition is to be written in the data processing module of the hardware circuit in the measurement system.

The data further show that the voltage amplitude has slight errors, which are caused by errors of the device module, and data conversion errors, among others, in a simulation environment. The inductance exists in loops with slightly declinational phases. Results show that theoretical values acquired by a non-contact voltage transducer are highly consistent with measured ones.

## 3. Simulation 

**Figure 3:**
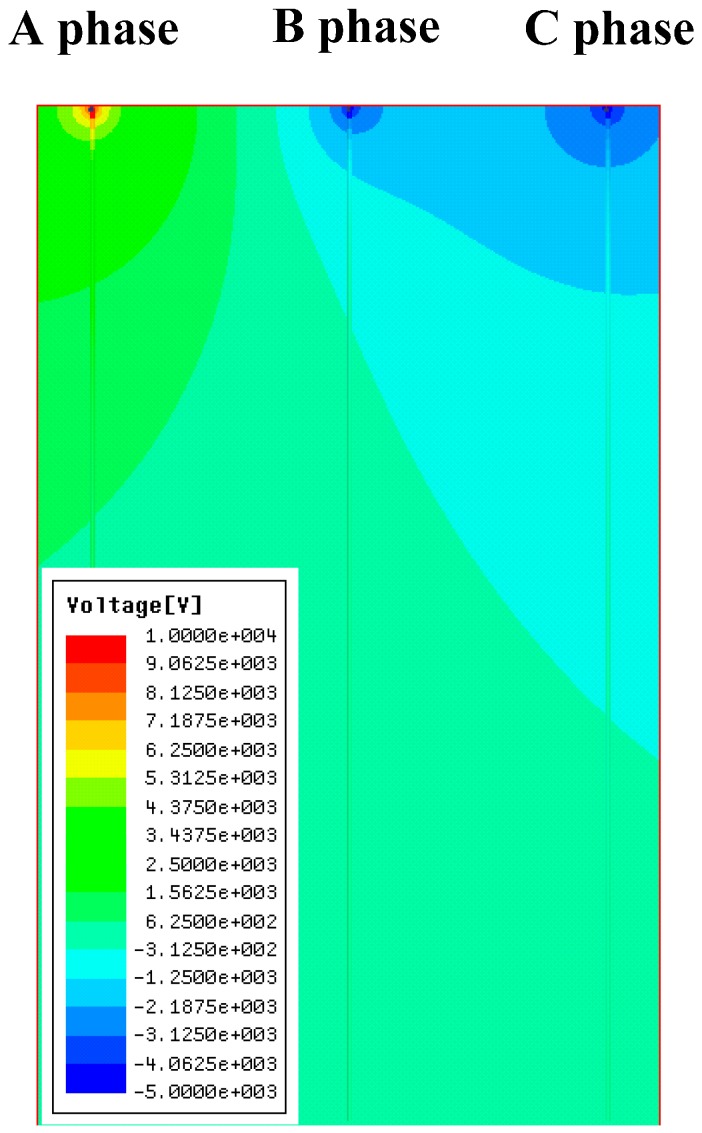
Voltage distribution of three-phase transmission line under the 10 kV voltage level.

In this paper, Ansoft software is used to implement the simulation of the electric field distribution under the transmission line. The results of the simulation are compared with the results of the subsequent measurement system. The model parameters of the three-phase transmission line to be established are listed in the following Table 3 and the simulation results are shown in the Figure 3.

**Figure 4:**
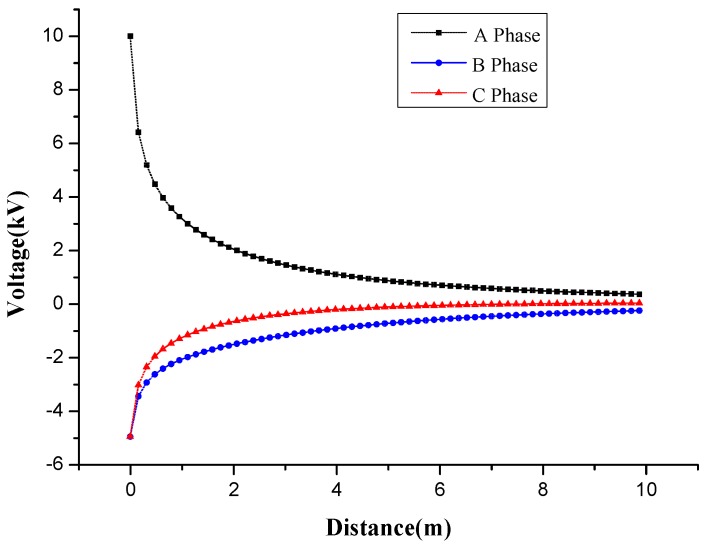
Voltage value under three phase transmission lines.

A line is drawn under each phase of the transmission line. The voltage value is checked at each point of the three lines, as shown in Figure 4.

## 4. Measurement System

**Figure 5:**
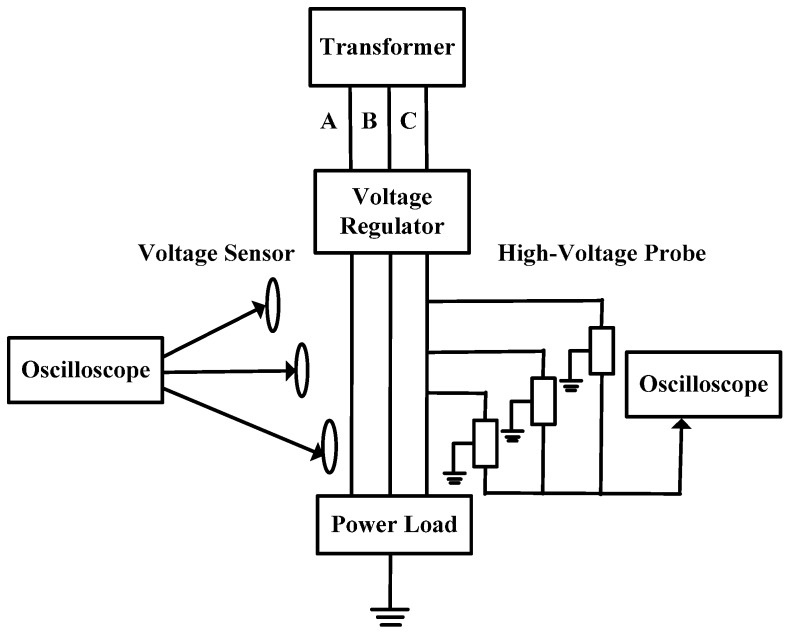
Structure of measurement system.

The voltage measurement system of three-phase D-dot transducer as show in Figure 5 is composed of two parts, namely, parts that respectively measure the hardware circuits and the PC-port software program. Based on the condition that the distance between conductor and transducer is not smaller than the distance between conductors, the composite electric field is inversely substituted into the algorithm for a voltage solution after decomposition, which can be achieved by the measurement system.

**Figure 6:**
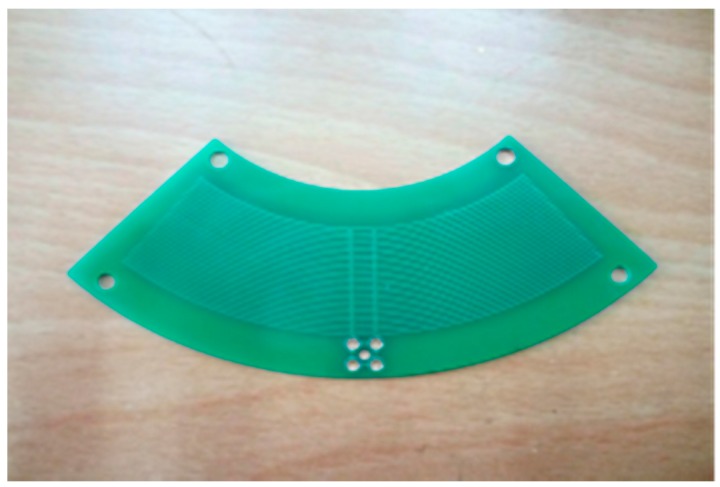
PCB of D-dot.

The D-dot electric field transducer is made into the form of a PCB (Printed Circuit Board) in Figure 6. The top layer and the bottom layer of the PCB are respectively provided with a plurality of annular electrodes with different radii, and the annular electrodes are respectively connected in parallel and have the same potential.

Voltage signals measured by the D-dot voltage transducer are processed for pre-amplification and electrical-level lifting by an analog signal processing module. After the treatment, a single-chip carries out high-speed collection and AD converter for the treated analog signals. Then the acquired digital signals are delivered to a WIFI wireless communication module according to the UART (Universal Asynchronous Receiver/Transmitter) Communication Protocol. The signals are then transformed to the UDP (User Datagram Protocol) protocol format and transferred to a data acceptance unit in the PC-port software by wireless networks.

After the data measured by the transducer is received by the data acceptance unit in a PC-port software part, iterative computations are executed by a digital signal processing unit to restore voltage waveforms of three phases. Moreover, the discrete signal collected by the sensor is converted into a continuous signal through the AD conversion, and the synthetic field decomposition algorithm proposed in the theoretical analysis is utilized. Then, the voltage waveform analysis unit analyzes this voltage signal to obtain information such as its voltage amplitude, frequency and degree of distortion. The waveform record unit performs the waveform storage for follow-up consultation and usage.

**Figure 7:**
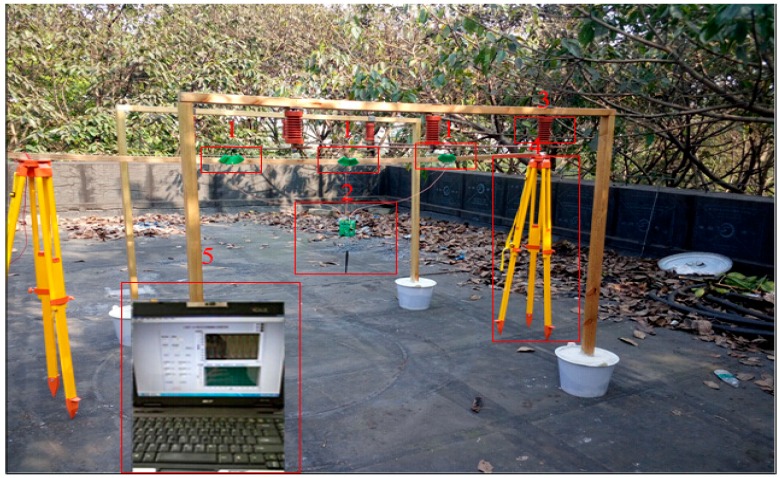
Sketch map of measuring three-phase voltage.

To test the D-dot transducer and the working performance of its measurement system, the three-phase experimental platform is established to measure the results and analyze the electric field environment of the 10 kV voltage power line transmission line. The measurement spot of the voltage transducer is shown in Figure 7. In Figure 7, number 1 represents the three-phase voltage transducers; they are installed under each of the three transmission lines respectively. Number 2 refers to the signal sample and the process circuit, hanging over it is a wireless transmission module. Number 3 is standard voltage transformer. Number 4 indicates support of the measurement system, and number 5 represents the host computer.

After the voltage regulator, the three-phase alternating current is loaded to three-phase transmission line after boosting the standard voltage transformer. The transducer is installed right under the transmission line and measuring circuits are connected to the transducer by signal lines. Collected data are then sent to the host computer by wireless module and the upper computer adopts the LabVIEW software for programming. Following the UDP Communication Protocol, data are received and transferred to corresponding iterative calculation module to a finally acquire the three-phase voltage waveform.

**Figure 8:**
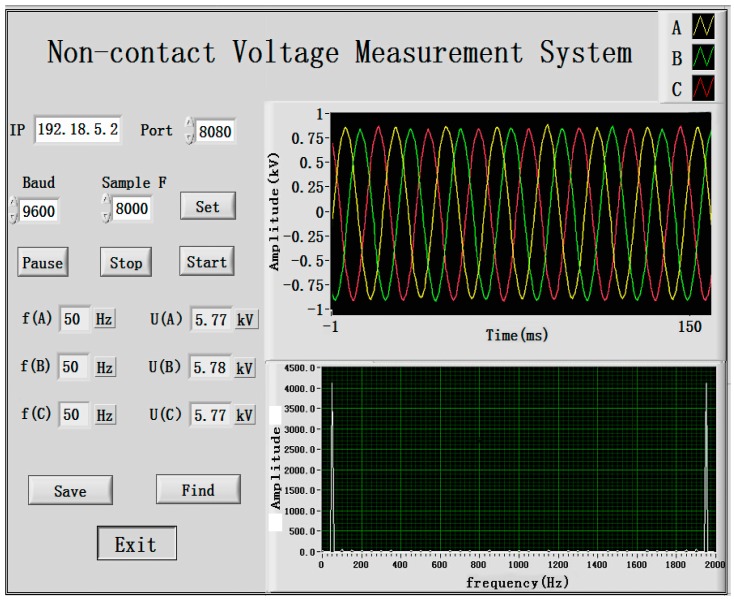
Interface of display on PC.

As shown in Figure 8, the display interface of LabVIEW is represented with an effective value of the measured-phase voltage, 5.8 kV, and a peak value of 8.2 kV. In the left side of the figure, the communication setting as well as the frequency and effective value of the three-phase voltage are illustrated. In the host computer section, the curves with colors of yellow, green and red are represented as domain waveforms of the three-phase voltage, whereas the lower part shows the analysis chart of the frequency spectrum.

## 5. Experiment

The experimental part of the article will be analyzed from two aspects, according to the IEC60044-7 Standard. The results of the two aspect experiment determine whether the performance of the transducer can meet the requirements of the actual application.

### 5.1. Steady-State Error Experiment

The steady-state error experiment is implemented in the built experimental platform. According to IEC60044-7 Standard, the value of the three-phase voltage is adjusted during the experiment. The proportion of the rated voltage is 10%, 20%, 40%, 60%, 80%, 100% and 120% respectively, using the high-voltage detection head and the non-contact voltage transformer for measurement. In addition, the high-voltage detection head is connected to a three-phase conductor and the oscilloscope is adopted to synchronously measure voltage. Finally, data in two different voltage measuring points are fit to acquire a comparison of the linearity between the high-voltage detection head and the experimental system and to obtain the error ratio of the voltage.

The detection results of High-voltage probe are represented by U_H_. Output voltages of the transducer are sent to the computing unit, which exist based on Equations (1)–(8) to acquire measured voltage U_M_ by inverse substitution. Dynamic range and linearity of the transducer are measured and results are shown in Table 4. 

**Figure 9:**
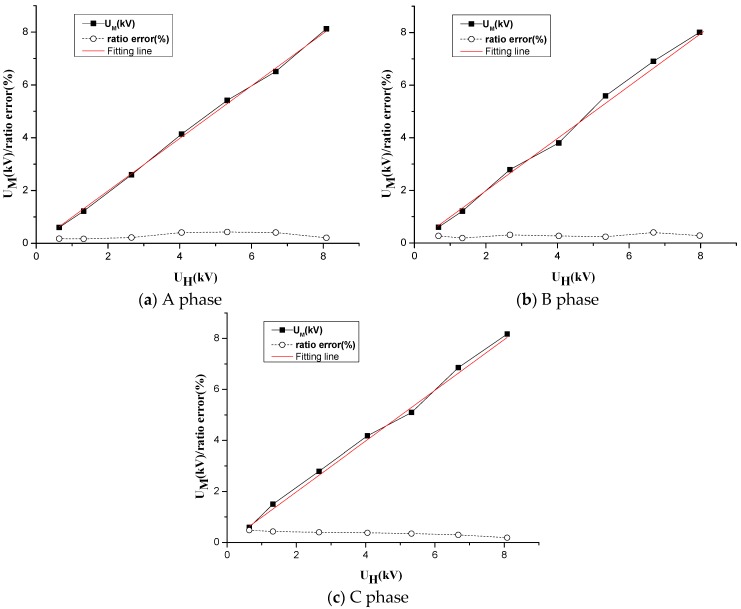
Calibration and ratio curve of the sensor and the HV-probe.

Based on the obtained detected data, a comparative experiment, the calibration curve and the error curve of the output voltage of the transducer and high-voltage detection head are shown in Figure 9.

### 5.2. Sensor Accuracy Test

When the initial phase of three-phase transmission line is 0, we use sensors to measure different positions under the transmission line to obtain their voltage values, and simultaneously extract voltage values from the Ansoft simulation results at the same point. We then contrast the two voltage values. The solid triangle represents the simulation results and the hollow triangle represents the result of the measurement as shown in the figure below. 

### 5.3. Result Analysis

Our comparison and analysis of the results measured by the high-voltage detection head and the transducer, are as follows.

(1) As shown in the LabVIEW display from Figure 8, the three-phase waveform is close to a standard sinusoidal waveform with a relatively small degree of distortion, which represents a three-phase measuring system that can realize synchronous acquisition by multipath signals.
(2) When the three-phase voltage changed within range of 10%–120% of the rated voltage, the specific value error of the electric-field coupling voltage transducer is ε% < 0.5%. The measured voltage in Figure 9 was fitted once with a goodness of fit of 0.9988, 0.9989 and 0.9993, proving that the transducer has a relatively good linearity in all voltage measuring points.

The measured results of the software simulation and transducer are compared and analyzed. 

**Figure 10:**
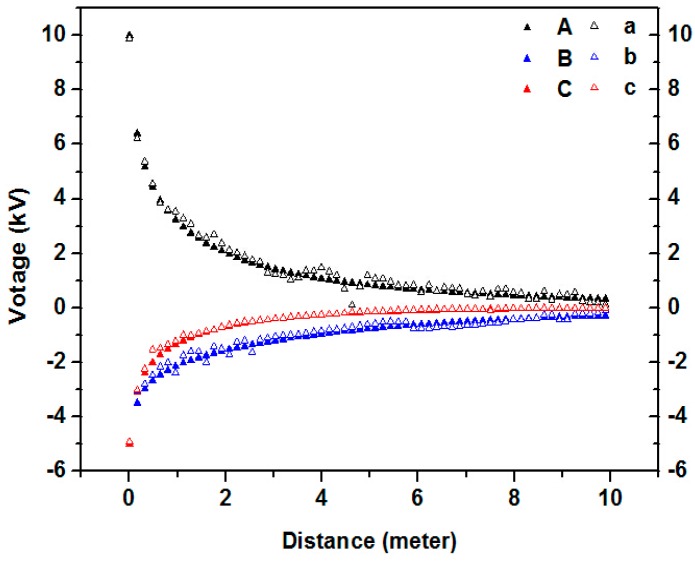
Comparison of simulation and measurement results.

In the comparative curve in Figure 10, the values measured by the transducer are in accordance with the change trend of simulation graphics and these two curves are extremely close to each other, which means that the measurement with the transducer is of relatively high precision.

From the two experiments above, we observe that the measured results have some errors and the error becomes more significant with an increase in the measured distance. This observation is because the boundary conditions adopted in the simulation calculation are ideal, but the conductor length, sag and transducer location all deviated from ideal conditions, thus largely affecting precision.

## 6. Conclusions

The three-phase D-dot voltage transducer measuring system was constructed and tested. With the aim of addressing the composite electric field problem during the measurement process of a three-phase transmission line, we proposed a decomposition method and verified its feasibility with experiments. The measuring system can realize synchronous acquisition by multipath signals and successfully meet the accuracy class of the 0.5-level voltage transformer used for measurement when the rated voltage is within the range of 10%–120%. Furthermore it has relatively good linearity across all voltage measuring points.

The non-contact voltage measuring system of a three-phase field-type transducer further promotes non-contact voltage measuring work in electric power projects. In cases where the measuring precision requirement is not high, the measuring system can achieve a rapid, convenient and safe measurement, which has relatively high engineering value in measuring electric parameters of distribution automation. In current projects, influences of tower sag and locations places of multiple transducers will result in errors of the voltage measurement in an experimental system. Thus, for future studies, the complexity degree and algorithm precision of the model should be improved to gradually resolve this problem.

## Figures and Tables

**Table 1:** Measurement results of electric field intensity under different voltages.

Voltage (kV)	E*_A_* (v/m)	ε*_A_* (v/m)	$\varphi_{u}$ (°)	E*_B_* (v/m)	ε*_B_* (v/m)	$\varphi_{u}$ (°)	E*_C_* (v/m)	ε*_C_* (v/m)	$\varphi_{u}$ (°)
1	119.7	13.9	2.98	68.7	6.9	1.68	66.7	7.0	1.66
2	337.5	28.1	2.65	191.5	11.1	1.55	181.4	13.1	1.53
3	457.8	35.7	2.07	246.1	16.7	1.47	245.2	14.2	1.41
4	560.9	38.9	1.78	290.3	18.8	1.38	277.3	15.6	1.35
5	684.2	40.4	1.23	390.6	22.4	1.29	329.5	19.8	1.28
6	895.3	42.9	1.05	495.5	25.9	1.25	395.8	22.5	1.23
7	933.6	46.5	0.98	533.5	28.7	1.19	413.5	26.4	1.20
8	989.5	47.8	0.81	559.7	29.2	0.98	459.1	27.2	0.94
9	1005.7	49.2	0.58	583.5	30.3	0.94	495.2	29.3	0.84
10	1123.5	50	0.34	616.2	30.5	0.82	521.1	31.5	0.79
11	1322.3	54.1	0.30	672.3	31.1	0.77	587.3	33.1	0.74
12	1436.4	55.2	0.23	756.4	33.6	0.53	668.7	36.3	0.63

**Table 2:** Relative amplitude error.

Voltage (kV)	ε_A_%	ε_B_%	ε_C_%
1	0.42	−0.38	−0.38
2	0.38	−0.37	−0.31
3	0.39	−0.30	−0.27
4	0.31	−0.26	0.13
5	0.25	−0.21	0.23
6	0.26	−0.18	0.21
7	−0.13	−0.11	0.35
8	−0.19	0.18	0.32
9	−0.22	0.29	0.38
10	−0.30	0.31	0.41
11	−0.35	0.34	0.43
12	−0.41	0.36	0.44

**Table 3:** Parameters of the simulation model.

Phase	Radius (mm)	X-Coordinates (m)	V (kV)	Phase (°)	Distance	Material
A	3.34	0.185	10	0	1.8	aluminum
B	3.34	0.540	10	120	1.8	aluminum
C	3.34	0.903	10		1.8	aluminum

**Table 4:** Accuracy test of three-phase D-dot voltage transformer.

Phase	Voltage/kV	U_H_/kV	U_M_/kV	Ratio Error/%
A	1	0.64	0.604	0.18
	2	1.322	1.221	0.17
	4	2.655	2.599	0.22
	6	4.051	4.139	0.41
	8	5.323	5.416	0.43
	10	6.676	6.508	0.41
	12	8.087	8.127	0.21
B	1	0.673	0.601	0.27
	2	1.341	1.212	0.19
	4	2.671	2.791	0.31
	6	4.036	3.802	0.27
	8	5.343	5.595	0.24
	10	6.683	6.909	0.4
	12	7.976	8.009	0.28
C	1	0.641	0.596	0.48
	2	1.322	1.232	0.43
	4	2.655	2.592	0.4
	6	4.051	3.979	0.38
	8	5.323	5.294	0.35
	10	6.676	6.603	0.3
	12	8.087	8.037	0.19
